# Superior antigen-specific CD4^+^T-cell response with AS03-adjuvantation of a trivalent influenza vaccine in a randomised trial of adults aged 65 and older

**DOI:** 10.1186/1471-2334-14-425

**Published:** 2014-07-30

**Authors:** Robert B Couch, José M Bayas, Covadonga Caso, Innocent Nnadi Mbawuike, Concepción Núñez López, Carine Claeys, Mohamed El Idrissi, Caroline Hervé, Béatrice Laupèze, Lidia Oostvogels, Philippe Moris

**Affiliations:** Department of Molecular Virology and Microbiology, Baylor College of medicine, Houston, Texas USA; Centro de Vacunación de Adultos, Servicio de Medicina Preventiva y Epidemiología, Hospital Clínic, Barcelona, Spain; Servicio de Prevención de Riesgos Laborales, Hospital Clínico San Carlos, Madrid, Spain; Servicio de Prevención Hospital Universitario La Paz, Madrid, Spain; GlaxoSmithKline Vaccines, Wavre, Belgium; GlaxoSmithKline Vaccines, Rixensart, Belgium; Department of Molecular Virology and Microbiology, Baylor College of Medicine, One Baylor Plaza, BCM280, Houston, TX 77030 USA

**Keywords:** Influenza vaccination, AS03 adjuvant system, T-cell response, Elderly, Immunosenescence, Cell-mediated immunity

## Abstract

**Background:**

The effectiveness of trivalent influenza vaccines may be reduced in older versus younger adults because of age-related immunosenescence. The use of an adjuvant in such a vaccine is one strategy that may combat immunosenescence, potentially by bolstering T-cell mediated responses.

**Methods:**

This observer-blind study, conducted in the United States (US) and Spain during the 2008–2009 influenza season, evaluated the effect of Adjuvant System AS03 on specific T-cell responses to a seasonal trivalent influenza vaccine (TIV) in ≥65 year-old adults.

Medically-stable adults aged ≥65 years were randomly allocated to receive a single dose of AS03-adjuvanted TIV (TIV/AS03) or TIV. Healthy adults aged 18–40 years received only TIV. Blood samples were collected on Day 0, Day 21, Day 42 and Day 180. Influenza-specific CD4^+^ T cells, defined by the induction of the immune markers CD40L, IL-2, IFN-γ, or TNF-α, were measured in *ex vivo* cultures of antigen-stimulated peripheral blood mononuclear cells.

**Results:**

A total of 192 adults were vaccinated: sixty nine and seventy three ≥65 year olds received TIV/AS03 and TIV, respectively; and fifty 18 - 40 year olds received TIV. In the ≥65 year-old group on Day 21, the frequency of CD4^+^ T cells specific to the three vaccine strains was superior in the TIV/AS03 recipients to the frequency in TIV (*p* < 0.001). On Days 42 and 180, the adjusted-geometric mean specific CD4^+^ T-cell frequencies were also higher in the TIV/AS03 recipients than in the TIV recipients (*p* < 0.001). Furthermore, the adjusted-geometric mean specific CD4^+^ T-cell frequencies were higher in the ≥65 year-old recipients of TIV/AS03 than in the18 - 40 year old recipients of TIV on Days 21 (*p* = 0.006) and 42 (*p* = 0.011).

**Conclusion:**

This positive effect of AS03 Adjuvant System on the CD4^+^ T-cell response to influenza vaccine strains in older adults could confer benefit in protection against clinical influenza disease in this population.

**Trial registration:**

(Clinicaltrials.gov.). NCT00765076.

**Electronic supplementary material:**

The online version of this article (doi:10.1186/1471-2334-14-425) contains supplementary material, which is available to authorized users.

## Background

Immune responses to influenza vaccination in older adults tend to be weaker than in younger adults and this has been attributed to the decline in immune function associated with advancing age, termed immunosenescence [1–6]. Strategies to improve vaccine immunogenicity in older adults include vaccination with a higher dose of antigen [7, 8] and the use of adjuvants [6]. However, conventional adjuvants based on aluminium salt do not typically improve immunogenicity of influenza vaccines [6, 9].

Adjuvant System AS03 (GlaxoSmithKline; GSK) is one of a new generation of adjuvants including MF59 (Novartis) and AF03 (Sanofi Pasteur) that have been shown to have the potential to enhance the immunogenicity of influenza vaccines [10–19]. AS03 contains *α*-tocopherol and squalene in an oil-in-water emulsion [20] and has been shown to enhance the immunogenicity of seasonal and potential pandemic vaccines in older adults [19, 21, 22]. AS03 was in the A/California/7/2009(H1N1)pdm09 vaccine that was administered to a large number of people during the A(H1N1) pandemic [20]. Preclinical experiments suggest that AS03 enhances the adaptive responses to vaccine antigens by triggering a transient innate response local to the injection site [23].

Influenza vaccine immunogenicity is typically assessed by serum antibody responses in haemagglutination-inhibition (HI) tests [24, 25]. However, cell-mediated immunity is another and important arm of the adaptive immune response: reports by Murasko *et al.*[26] and McElhaney *et al*. [3, 27] suggest that in older adults, cell-mediated responses play a significant role in protection from influenza disease after vaccination. Also, cell-mediated responses have been correlated with limiting disease severity in healthy ≥18 year-old adults [28, 29]. Recently, an AS03-adjuvanted trivalent influenza vaccine has been found to be more efficacious in preventing A(H3N2)-associated influenza than the equivalent unadjuvanted vaccine in a phase 3 trial of older adults [30]. The main aim of our study was to evaluate the effect of TIV adjuvanted with AS03 on T-cell responses in ≥65 year-old adults.

## Methods

### Study vaccines and vaccination

The investigational trivalent inactivated split influenza vaccine adjuvanted with AS03 (TIV/AS03; lot, DFLEA012B) and control vaccine (TIV; *Fluarix*™^a^; lot, AFLUA348A) were 2008-2009 seasonal vaccines supplied by GSK, Dresden, Germany. Vaccinations were performed on Day 0. One dose of TIV/AS03 or TIV contained 15 μg Haemagglutinin (HA) from each of the three influenza strains A/Brisbane/59/2007(H1N1), A/Uruguay/716/2007 (an A/Brisbane/10/2007[H3N2]-like virus) and B/Brisbane/3/2007 (a B/Florida/4/2006-like virus) (total 45 μg HA). AS03 (B formulation) contained 5.93 mg *α*-tocopherol, 5.34 mg squalene and 2.43 mg polysorbate-80 and was supplied by GSK, Rixensart, Belgium. The vaccines were administered by intramuscular injection (23G and length suited to subject’s body mass) as 0.7 ml (TIV/AS03) or 0.5 ml (TIV) into the deltoid muscle at study entry (Day 0).

### Study design and participants

This randomised, observer blind controlled study (NCT-00765076) was conducted in the United States (US) and Spain between October 2008 and December 2009. The protocol, its amendments and other relevant study documentation were approved by the Institutional Review Board for Baylor College of Medicine and Affiliated Hospitals, the Comité Etico de Investigación Clínica del Hospital Clinic de Barcelona, the Comité Etico de Investigación Clínica del Hospital La Paz, and the Comité Etico de Investigación Clínica del Hospital Clínico San Carlos, and the study was conducted in accordance with good clinical practice guidelines, the Declaration of Helsinki and all applicable regulatory requirements. Eligible participants had provided written informed consent. They were non-pregnant healthy adults aged 18-40 years or medically-stable adults aged ≥65 years at the time of the vaccination. Adults ≥65 years were randomly allocated (1:1) to receive TIV/AS03, (group TIV/AS03[≥65]) or TIV (group TIV[≥65]). Adults 18-40 years received TIV only (group TIV[18–40]) and the study was open for this group. As the appearances of the investigational and control vaccines were different, the study was observer-blind for the ≥65-years groups with vaccinations performed by study personnel not involved in the assessment of immunogenicity or safety/reactogenicity. A randomisation list was generated by the sponsor using SAS version 9.2 (SAS Institute Inc., NC, USA) and used to number the vaccines. A randomisation blocking scheme was used (respecting the treatment allocation 3:3:2 ratio for the three study groups) and a treatment number uniquely identified the vaccine to be administered to each subject. The treatment allocation for the ≥65 year old subjects at the investigator site was performed using an internet-based system. The randomisation algorithm used a minimisation procedure accounting for centre and age strata 65-74 years and ≥75 years.

### Study endpoints

The primary immunological endpoint was the frequency of antigen-induced CD4^+^ T-cells specific for the three combined vaccine influenza strains measured at Day 21, in the groups of ≥65 year olds. Specific CD4^+^ T cells were identified as CD4^+^ T cells which express two or more of the following antigen-response markers after short-term (20 hours) *ex vivo* stimulation: the CD40L activation marker, and the cytokines IL-2, IFN-γ, or TNF-α. The secondary immunological endpoints were measured at Days 0, 21, 42 and 180 in all groups and included the frequencies of vaccine-strain-specific CD4^+^ T-cells, and vaccine-strain-specific serum HI titres. Safety and reactogenicity were assessed as secondary endpoints. Exploratory endpoints included, in all subjects, vaccine strain-specific neutralising antibody titres, and the frequencies of vaccine-strain specific CD8^+^ T cells that expressed two or more of the markers, CD40L, IL-2, IFN-γ, or TNF-α; and, in a subset of subjects, the frequencies of vaccine-strain-specific CD4^+^ or CD8^+^ T cells that expressed Granzyme B and IFN-γ and/or IL-2, and, in those who were cytomegalovirus (CMV)-seropositive at baseline, the frequencies of CMV-specific T cells that expressed Granzyme B and IFN-γ and/or IL-2.

### Sample size

Sample sizes were set at 75 for each of the two ≥65 year groups and at 50 in the TIV(18–40) group assuming five subjects/per group would be non-evaluable. Seventy subjects in each of the ≥65 year age groups would give 90% power to demonstrate superiority of TIV/AS03 over TIV in vaccine strain-specific CD4^+^ T-cell responses, assuming a 1.5-fold greater response in the TIV/AS03 recipients compared with the TIV recipients (based on unpublished observations), and with a coefficient of variation of 100% and a type I error of 5% (1-sided). Forty five subjects in the TIV(18–40) group was calculated to give 80% power to demonstrate non-inferiority of TIV/AS03 in the ≥65 year age group compared with TIV in the 18–40 year age group in terms of vaccine strain-specific CD4^+^ T-cell responses assuming no difference in this response to that for the TIV/AS03(≥65) group and with a coefficient of variation of 100%, a type I error of 5% and a non-inferiority margin of 1.5 fold. However, this non-inferiority evaluation was not used as a confirmatory objective.

### *Ex vivo*short-term T-cell re-stimulation assay

Influenza-specific T-cell responses were assessed using a previously described validated method [14, 31] adapted from Maecker *et al.*[32]. Peripheral blood mononuclear cells (PBMCs) were stimulated for two hours by the split vaccine strain antigens in the presence of co-stimulatory antibodies to CD28 and CD49d [33]. Brefeldin A was added for a subsequent 18 hours incubation to promote intracellular accumulation of cytokines [33]. Cells were stained using fluorochrome-conjugated antibodies before enumeration by flow cytometry. T cells were identified by positive expression of CD3. CD4^+^ or CD8^+^ T cells were typed as specific when they expressed two or more immune markers among CD40L, IFN-γ, IL-2, TNF-α. In some of the exploratory objectives, CD4^+^ or CD8^+^ T cells were also typed as specific when they expressed Granzyme B and IFN-γ or IL-2.

CMV-specific T-cell responses (in the Spain-enrolled subset) were identified by the expression of Granzyme B and IFN-γ or IL-2, using the same *ex vivo* method except that PBMCs were stimulated by incubation with split-CMV virus (to assess bystander activation of T cells [34, 35]).

### HI assay

Sera were analysed in a micro-titre HI assay as described previously [36] with the vaccine virus strains used as antigens. The serum titre was expressed as the reciprocal of the highest dilution that showed complete inhibition of haemagglutination. Seroconversion rate (SCR), seroprotection rate (SPR) and seroconversion factor (SCF) were defined according to regulatory criteria [24, 25].

### Neutralisation assay

Sera were subjected to heat treatment at 56°C for 30 minutes and then tested in triplicate as previously described [37]. A constant amount of virus was mixed with serial two-fold dilutions of serum samples, added to Madin-Darby Canine Kidney cell cultures and incubated for 7 days at 37°C. After incubation, virus replication was determined by haemagglutination of red blood cells. The 50% neutralisation titre of a serum was calculated by the Reed and Muench method [38].

### Safety/reactogenicity assessments

Injection site adverse events (AEs; ecchymosis, pain, redness and swelling) and systemic AEs (generalised/widespread arthralgia, fatigue, gastrointestinal symptoms, headache, generalised/widespread myalgia, shivering and fever) were solicited daily for 7 days after vaccination. Unsolicited AEs and their intensities were recorded for 21 days after vaccination. The intensities of ecchymosis, redness and swelling were graded as follows: Grade 1, >20– ≤ 50 mm; Grade 2, >50– ≤ 100 mm; and Grade 3, >100 mm. Daily body temperature was graded as follows: Grade 1, 38.0– < 38.5°C; Grade 2, 38.5– < 39.0°C; and Grade3, 39.0– ≤ 40.0°C. The intensities of other AEs, including unsolicited AEs were graded as follows: Grade 1, “easily tolerated” (“painful on touch” for injection site pain); Grade 2, “interferes with normal activity” (or “painful when limb is moved” for injection site pain); and Grade 3, “prevents normal activity” (or “considerable pain at rest” for injection site pain). Data on unsolicited medically-attended AEs were recorded up to Day 180. Data on serious adverse events (SAEs) and any cases of potential Immune Mediated Disease (pIMD, including diseases with clear autoimmune aetiology and other inflammatory and/or neurologic disorders with or without an autoimmune aetiology) were collected during the whole study period (1 year). An assessment of causality was made by the investigator for solicited systemic and unsolicited AEs, as well as for SAEs and pIMDs.

The use of medication during the study was recorded from Day 0 to Day 180, and was reviewed by the investigator for any potential relationship with a study measurement. The medication was considered as prophylactic when it was administered in the absence of any symptom and in anticipation of a reaction to the vaccination.

### Statistical analysis

The primary objective was to demonstrate in vaccinated subjects ≥65 years old, the superiority of TIV/AS03 over TIV at the 5% significance level (1-sided), by having the lower limit of the 90% confidence interval (CI) greater than 1 of the adjusted geometric mean ratio (GMR) of vaccine influenza strain-specific CD4^+^ T-cell frequencies at Day 21. Statistical analyses of the secondary and exploratory endpoints were descriptive. For the analyses of CD4^+^ T-cell responses between groups, a likelihood-based mixed effect ANCOVA model for repeated measurement was used to analyse the post-vaccination log-transformed frequencies of influenza-specific CD4^+^ T cells. The model included vaccine group, visit, and vaccine group by visit interactions as fixed effects and the pre-vaccination log-transformed frequency as covariate (resulting in the calculation of the *adjusted* geometric mean frequency [GMF]). An unstructured covariance matrix was used to account for the repeated measurements among subjects. The goodness of fit Bayesian Information Criteria statistic (SBIC) was used to assess the need of a separate covariance matrix for each treatment. At each time point and for each group, the adjusted geometric mean of post-vaccination specific CD4^+^ T-cell frequency was computed together with its 90% CI. The ratios of TIV/AS03(≥65) over TIV(≥65) or over TIV(18 - 40) were calculated with their 90% CIs. Computation of geometric mean titres (GMT) and SCFs, and their 95% CIs, used Student’s *t*-distribution on log_-_transformed data that was assumed to be normally distributed with unknown variance. Exact 95% CIs were calculated for SCRs, SPRs and the safety endpoints. Pearson correlation coefficients (*r*) were calculated on the log-transformed values of comparisons of fold-changes (Day 21 over Day 0) between vaccine strain-specific HI titres and CD4^+^ T-cell frequencies. *SAS* (version 9.2, SAS Institute Inc., NC, USA) was used for all computations. Titre values in the text are reported to two significant figures.

## Results

### Study population

**Figure 1:**
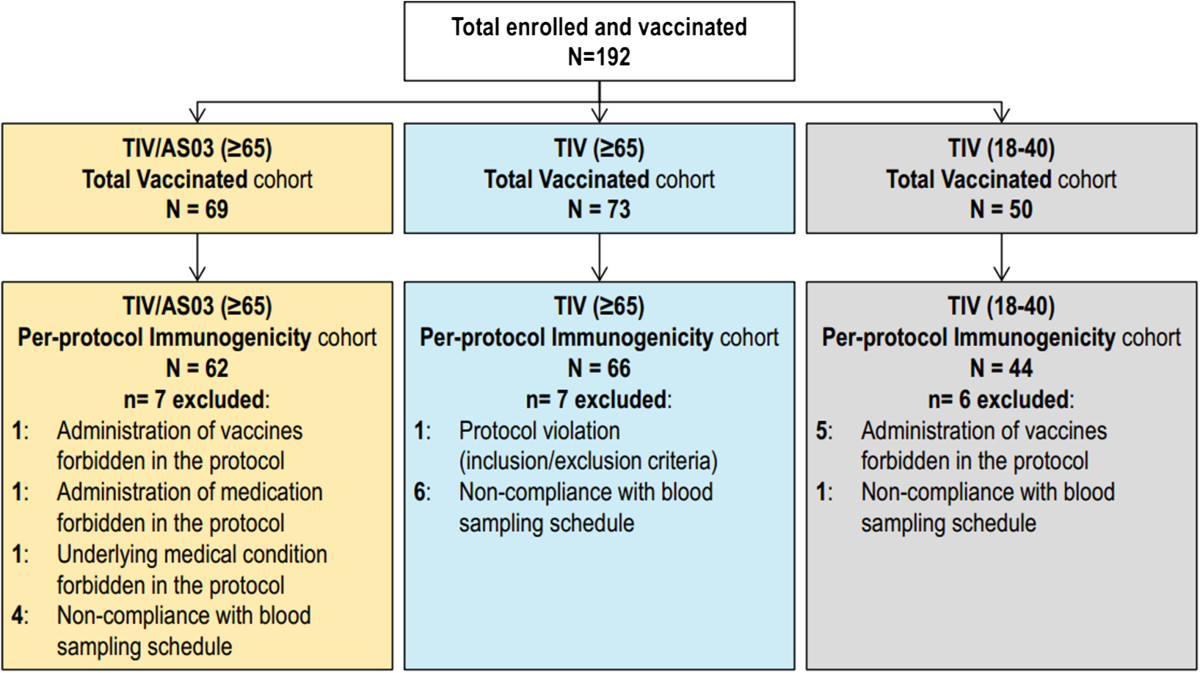
**The allocation and elimination of subjects during the course of the study.** The reasons for elimination from the Total Vaccinated cohort (TVC) to give the per protocol immunogenicity cohorts are described to the left of each box. Safety was assessed in the TVC.

A total of 192 adults were enrolled and, of the ≥65 year olds, 69 were vaccinated with TIV/AS03 and 73 were vaccinated with TIV. Fifty 18–40 year olds were also vaccinated with TIV (Figure 1). One hundred and six subjects were from Spain and 86 from the US. Two subjects did not complete the study, but were vaccinated. One subject (TIV/AS03[≥65] group) did not complete due to a fatal myocardial infarction; and the other subject (TIV[18 - 40] group) withdrew consent for a reason not related to any AE. Twenty subjects were excluded from the per protocol immunogenicity cohorts. In these per protocol cohorts, the mean ages at enrolment of the two ≥65 year groups were both 71 years and the mean age of the 18 - 40 years group was 26 years. The majority of subjects in all groups were White Caucasian (Table 1). With respect to the proportion of female subjects, there was an imbalance between the three groups (50%, 41% and 54% in the TIV/AS03[≥65], TIV[≥65] and TIV[18 - 40], respectively). In the three influenza seasons preceding the study, 71%, 78% and 48% of the subjects in the TIV/AS03(≥65), TIV(≥65) and TIV(18 - 40) groups, respectively, had received influenza vaccination.

**Table 1:** **Demographic characteristics of the per-protocol cohort**

Characteristic		TIV/AS03(≥65)	TIV(≥65)	TIV(18–40)	Total
		(N = 62)	(N = 66)	(N = 44)	(N = 172)
Age, months; mean (SD)		71.4 (5.5)	71.3 (5.3)	26.3 (5.1)	59.8 (20.4)
Gender; n (%)	Female	31 (50)	27 (41)	24 (55)	82 (48)
	Male	31 (50)	39 (59)	20 (46)	90 (52)
Race; n (%)	American Indian or Alaskan native	0 (0)	0 (0)	0 (0)	0 (0)
	African heritage/African American	0 (0)	0 (0)	0 (0)	0 (0)
	Asian–Central/S. Asian heritage	0 (0)	0 (0)	2 (4.5)	2 (1.2)
	Asian–E. Asian heritage	0 (0)	0 (0)	2 (4.5)	2 (1.2)
	Asian–Japanese heritage	0 (0)	0 (0)	0 (0)	0 (0)
	Asian–S. E. Asian heritage	0 (0)	1 (1.5)	1 (2.3)	2 (1.2)
	Native Hawaiian/pacific islander	0 (0)	0 (0)	0 (0)	0 (0)
	White - Arabic/N. African heritage	0 (0)	0 (0)	1 (2.3)	1 (0.6)
	White - Caucasian/European heritage	62 (100)	64 (97)	37 (84)	163 (95)
	Other	0 (0)	1 (1.5)	1 (2.3)	2 (1.2)

SD, standard deviation; TIV, trivalent influenza vaccine; TIV/AS03, AS03-adjuvanted trivalent influenza vaccine.

### Immunogenicity

On Day 21 in the ≥65 year old subjects, TIV/AS03 was superior to TIV by a factor of 1.64 (*p* < 0.001) in terms of the adjusted-GMR of frequencies of CD4^+^ T cells specific to the three (pooled) vaccine strains per 10^6^ CD4^+^ T cells (Table 2). On Days 42 and 180 in the ≥65 year old subjects, the adjusted-GMRs of vaccine strain-specific CD4^+^ T-cell frequencies in the TIV/AS03 group over the TIV group were 1.70 (*p* < 0.001) and 1.40 (*p* < 0.001), respectively. On Days 21 and 42, adjusted-GMRs of the specific CD4^+^ T-cell frequencies in the TIV/AS03(≥65) group over the TIV(18–40) group were 1.35 (*p* = 0.006) and 1.30 (*p* = 0.011), respectively, whereas on Day 180, the adjusted-GMR was 0.99 (*p* = 0.860).

**Table 2:** **Influenza-specific CD4+ T-cell responses to vaccination**

Day	CD4^+^T cells^a^producing at least two different markers (CD40L, IL-2, TNF-α, IFN-γ) / 10^6^CD4^+^T cells		
	Adjusted geometric mean^b^(90% CI; N)		
	TIV/AS03(≥65)	TIV(≥65)	TIV(18–40)
21	3634 (3134–4214; N = 62)	2222 (1954–2527; N = 58)	2683 (2425–2969; N = 43)
42	2873 (2523–3271; N = 61)	1688 (1523–1871; N = 60)	2217 (2001–2457; N = 44)
180	2234 (2011–2482; N = 60)	1601 (1443–1776; N = 60)	2265 (2108–2433; N = 42)
	**Ratio (90% CI;** ***p*** **-value)**		
	**TIV/AS03(≥65), TIV(≥65)**		**TIV/AS03(≥65), TIV(18–40)**
21	1.64 (1.35–1.99; *p* < 0.001)^c^		1.35 (1.13–1.62; *p* = 0.006)^d^
42	1.70 (1.44–2.00; *p* < 0.001)		1.30 (1.10–1.53; *p* = 0.011)
180	1.40 (1.21–1.61; *p* < 0.001)		0.99 (0.87–1.12; *p* = 0.860)

^a^After *in vitro* stimulation with split vaccine antigens from all three (pooled) strains.

^b^Note that in the ANCOVA model, only the CD4^+^ T-cell frequency post-vaccination was considered as a dependent variable. The pre-vaccination CD4^+^ T-cell frequency was considered as a covariate in the calculation of the *adjusted* geometric means.

^c^Primary study objective.

^d^Secondary study objective. TIV/AS03: AS03-adjuvanted trivalent influenza vaccine.

**Figure 2:**
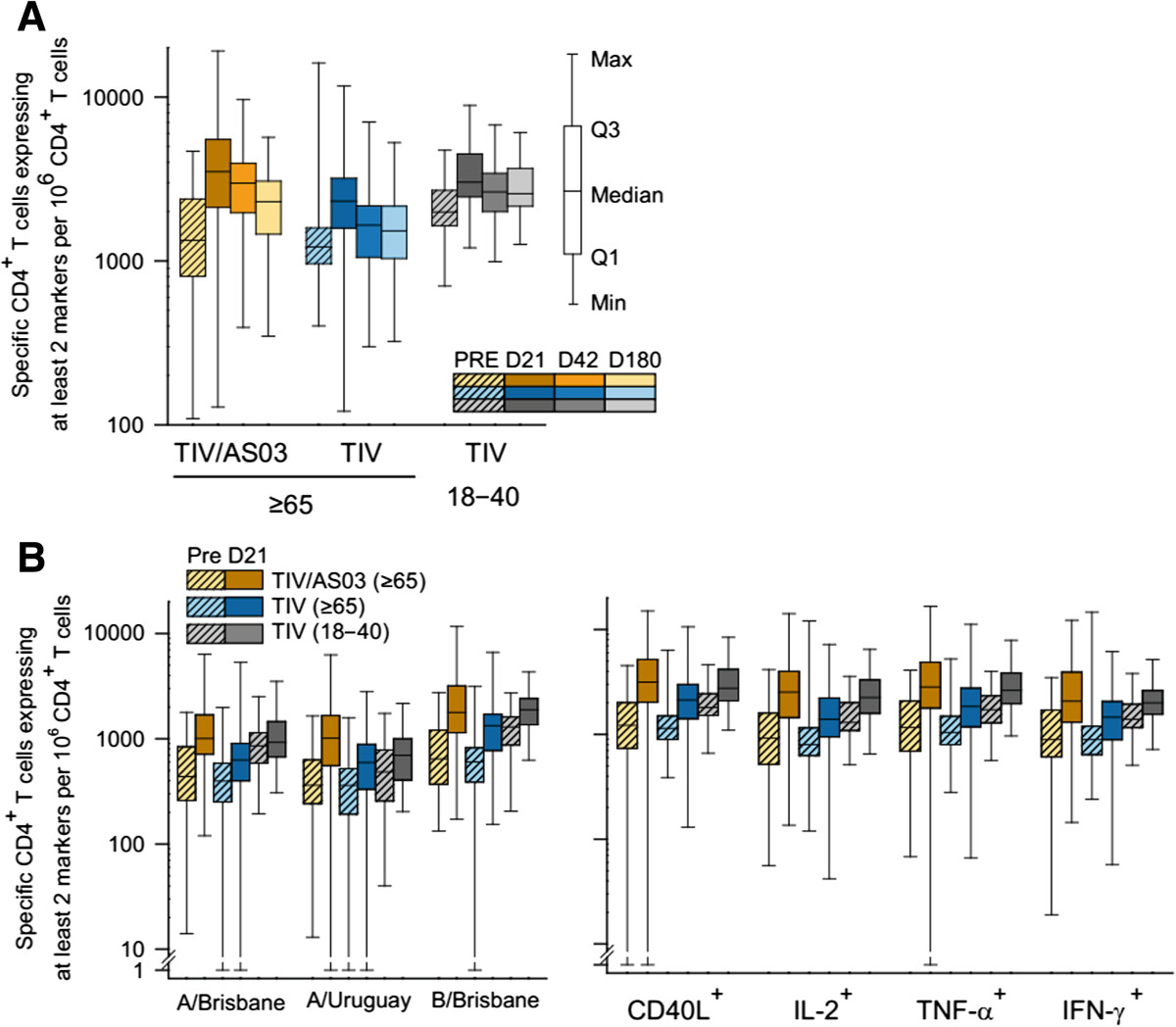
**Influenza-specific CD4** ^**+**^ **T-cell responses to vaccination in the immunogenicity cohort.** Box and whisker plots describing the frequency of CD4^+^ T cells **(A)** specific for the three (pooled) influenza vaccine strains and identified as expressing two or more immune markers among CD40L, IL-2, TNF-α and IFN-γ after a short term *in vitro* stimulation; (**B**-left) specific for each of the individual influenza vaccine strains and induced to express at least two immune markers; and (**B**-right) specific for the three (pooled) influenza vaccine strains and induced to express one defined immune marker (x-axis) and at least one other. The whiskers extend to the lowest (Min) and highest (Max) values; the box extends to the 1st quartile (Q1) and 3rd quartiles (Q3) in which the median is marked by a horizontal line.

Pre-vaccination, the median frequencies of (pooled) vaccine influenza strain-specific CD4^+^ T cells (per 10^6^ CD4^+^ T cells) were 1300 and 1200 in the TIV/AS03(≥65) and TIV(≥65) groups; whereas the median frequency was 2000 in the TIV(18–40) group. On Day 21, the respective median frequencies of these specific CD4^+^ T cells were 3500, 2300 and 3000, and on Day 180 they were 2300, 1500 and 2600 (Figure 2A). Each of the immune markers was detected in influenza-specific CD4^+^ T cells (Figure 2B) both before and after vaccination in the three groups. Each of the vaccine-strain antigens also stimulated specific CD4^+^ T-cell responses (Figure 2B). For A/Brisbane(H1N1), the pre-vaccination and Day 21 median frequencies in the TIV/AS03(≥65) group were 440 and 1000 respectively; whereas in the TIV(≥65), they were 400 and 630, respectively, and in TIV(18 - 40), they were 860 and 930, respectively. For A/Uruguay(H3N2), the corresponding median frequencies in the TIV/AS03(≥65) group were 360 and 1000, in the TIV(≥65) group, were 360 and 590, and in the TIV(18 - 40) group, were 480 and 700. For B/Brisbane, the corresponding median frequencies in the TIV/AS03(≥65) group were 650 and 1800, in the TIV(≥65) group, were 600 and 1300, and in the TIV(18 - 40) group, were 1300 and 1900. The relative proportions of the different influenza-specific CD4^+^ T-cell phenotypes appeared similar for the three groups (Figure 2B and Table 3). These phenotypes were mainly polyfunctional, and the most prevalent was CD40L^+^ IL-2^+^ IFN-γ^+^ TNF-α^+^ (Table 3). About half of the CD4^+^ T cells were IFN-γ^+^. The frequencies of a particular vaccine-strain specific CD4^+^ T-cell phenotype subset relative to the all vaccine-strain specific CD4^+^ T-cells appeared similar pre- and post-vaccination.

**Table 3:** **CD4** ^**+**^ **T-cell responses in terms of different immune marker expression profiles**

Immune-related marker expression (+/-)				Geometric mean frequencies^a^(percentages relative to totals) of CD4^+^T cells producing immune-related markers/10^6^CD4^+^T cells^b^			
**IFN-γ**	**TNF-α**	**IL-2**	**CD40L**	**Day 0**	**Day 21**	**Day 42**	**Day 180**
				**TIV/AS03(≥65)**			
**+**	**+**	**+**	**+**	490 (44)	1075 (31)	937 (37)	670 (37)
**+**	**-**	**+**	**+**	112 (10)	441 (13)	327 (13)	213 (12)
**-**	**+**	**+**	**+**	171 (15)	425 (12)	355 (14)	284 (16)
**-**	**-**	**+**	**+**	114 (10)	356 (10)	308 (12)	172 (10)
**+**	**+**	**-**	**+**	27 (2)	179 (5)	108 (4)	40 (2)
**+**	**-**	**-**	**+**	26 (2)	270 (8)	134 (5)	62 (3)
**-**	**+**	**-**	**+**	32 (3)	106 (3)	67 (3)	50 (3)
**-**	**-**	**-**	**+**	85 (8)	259 (8)	139 (5)	178 (10)
**+**	**+**	**+**	**-**	19 (2)	61 (2)	48 (2)	54 (3)
**+**	**-**	**+**	**-**	6 (1)	35 (1)	17 (1)	13 (1)
**-**	**+**	**+**	**-**	3 (0)	18 (1)	12 (0)	6 (0)
**-**	**-**	**+**	**-**	8 (1)	38 (1)	28 (1)	11 (1)
**+**	**+**	**-**	**-**	4 (0)	28 (1)	17 (1)	12 (1)
**+**	**-**	**-**	**-**	23 (2)	149 (4)	61 (2)	20 (1)
**-**	**+**	**-**	**-**	3 (0)	11 (0)	3 (0)	4 (0)
**Total**				**1124 (100)**	**3451 (100)**	**2562 (100)**	**1788 (100)**
				**TIV(≥65)**			
**+**	**+**	**+**	**+**	554 (45)	779 (33)	598 (38)	463 (34)
**+**	**-**	**+**	**+**	115 (9)	340 (15)	222 (14)	157 (12)
**-**	**+**	**+**	**+**	187 (15)	327 (14)	229 (15)	209 (16)
**-**	**-**	**+**	**+**	127 (10)	278 (12)	174 (11)	114 (8)
**+**	**+**	**-**	**+**	35 (3)	82 (3)	43 (3)	31 (2)
**+**	**-**	**-**	**+**	25 (2)	144 (6)	54 (3)	39 (3)
**-**	**+**	**-**	**+**	51 (4)	49 (2)	44 (3)	37 (3)
**-**	**-**	**-**	**+**	70 (6)	190 (8)	109 (7)	198 (15)
**+**	**+**	**+**	**-**	12 (1)	35 (1)	28 (2)	41 (3)
**+**	**-**	**+**	**-**	8 (1)	17 (1)	9 (1)	9 (1)
**-**	**+**	**+**	**-**	4 (0)	12 (1)	8 (1)	6 (0)
**-**	**-**	**+**	**-**	10 (1)	27 (1)	16 (1)	9 (1)
**+**	**+**	**-**	**-**	5 (0)	11 (0)	6 (0)	9 (1)
**+**	**-**	**-**	**-**	17 (1)	51 (2)	25 (2)	20 (2)
**-**	**+**	**-**	**-**	3 (0)	4 (0)	3 (0)	4 (0)
**Total**				**1222 (100)**	**2344 (100)**	**1568 (100)**	**1347 (100)**
**Immune-related marker expression (+/-)**				**Geometric mean frequencies** ^**a**^ **(percentages relative to totals) of CD4** ^**+**^ **T cells producing immune-related markers/10** ^**6**^ **CD4** ^**+**^ **T cells** ^**b**^			
**IFN-γ**	**TNF-α**	**IL-2**	**CD40L**	**Day 0**	**Day 21**	**Day 42**	**Day 180**
				**TIV(18–40)**			
**+**	**+**	**+**	**+**	832 (42)	1160 (32)	1007 (36)	863 (34)
**+**	**-**	**+**	**+**	220 (11)	530 (14)	402 (14)	290 (11)
**-**	**+**	**+**	**+**	239 (12)	347 (9)	283 (10)	265 (10)
**-**	**-**	**+**	**+**	182 (9)	389 (11)	306 (11)	256 (10)
**+**	**+**	**-**	**+**	69 (4)	145 (4)	136 (5)	87 (3)
**+**	**-**	**-**	**+**	50 (3)	230 (6)	139 (5)	122 (5)
**-**	**+**	**-**	**+**	56 (3)	101 (3)	87 (3)	53 (2)
**-**	**-**	**-**	**+**	181 (9)	340 (9)	194 (7)	276 (11)
**+**	**+**	**+**	**-**	67 (3)	121 (3)	95 (3)	199 (8)
**+**	**-**	**+**	**-**	13 (1)	61 (2)	31 (1)	46 (2)
**-**	**+**	**+**	**-**	7 (0)	19 (1)	11 (0)	7 (0)
**-**	**-**	**+**	**-**	13 (1)	92 (3)	25 (1)	11 (0)
**+**	**+**	**-**	**-**	9 (0)	34 (1)	22 (1)	25 (1)
**+**	**-**	**-**	**-**	19 (1)	89 (2)	69 (2)	44 (2)
**-**	**+**	**-**	**-**	4 (0)	8 (0)	5 (0)	4 (0)
**Total**				**1961 (100)**	**3669 (100)**	**2814 (100)**	**2548 (100)**

^a^Note that the geometric mean frequencies are not adjusted (see Table 2).

^b^After *in vitro* stimulation with split vaccine antigens from all three (pooled) strains.

TIV, trivalent influenza vaccine; TIV/AS03, AS03-adjuvanted trivalent influenza vaccine.

**Figure 3:**
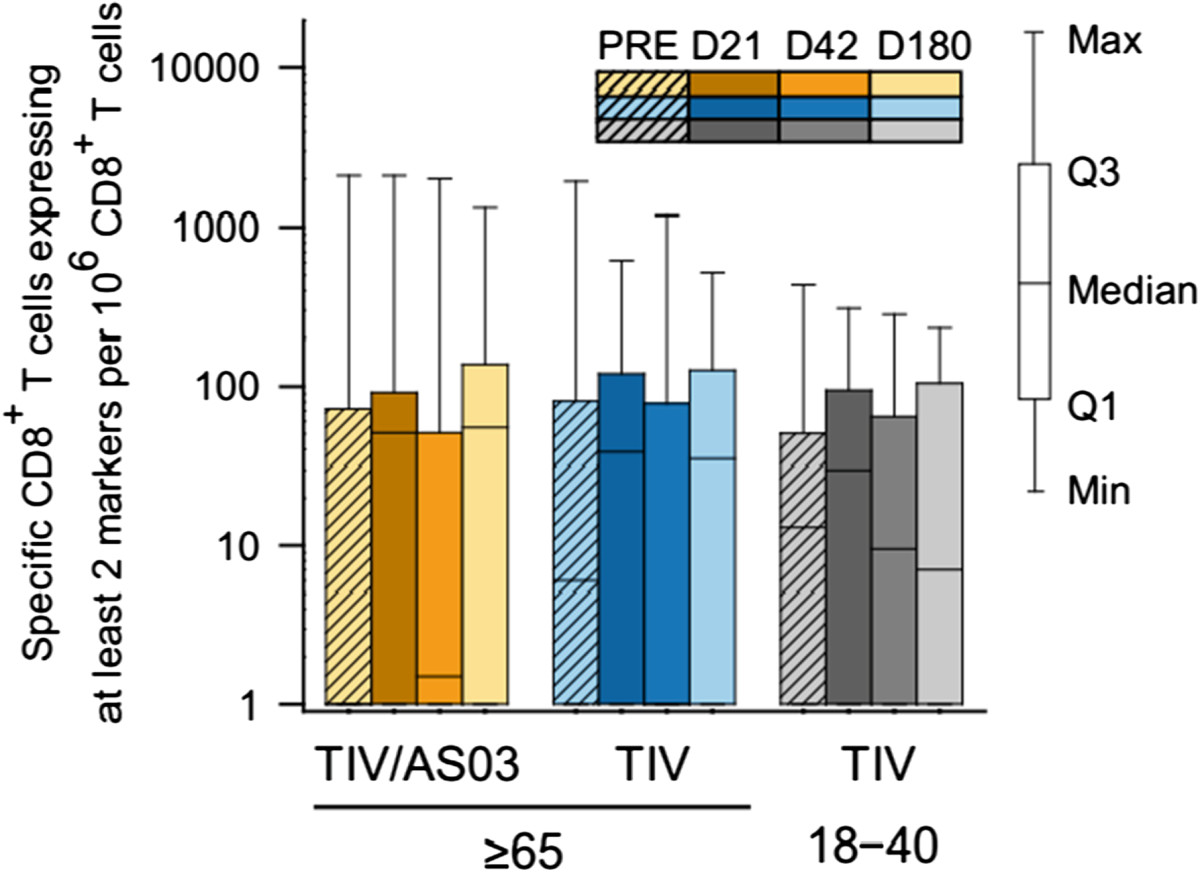
**Influenza-specific CD8** ^**+**^ **T-cell responses to vaccination in the per protocol immunogenicity cohort.** Box and whisker plots describing the frequency of CD8^+^ T cells specific for the three (pooled) influenza vaccine strains and induced to express at least two immune markers. The whiskers extend to the lowest (Min) and highest (Max) values; the box extends to the 1st quartile (Q1) and 3rd quartiles (Q3) in which the median is marked by a horizontal line.

**Figure 4:**
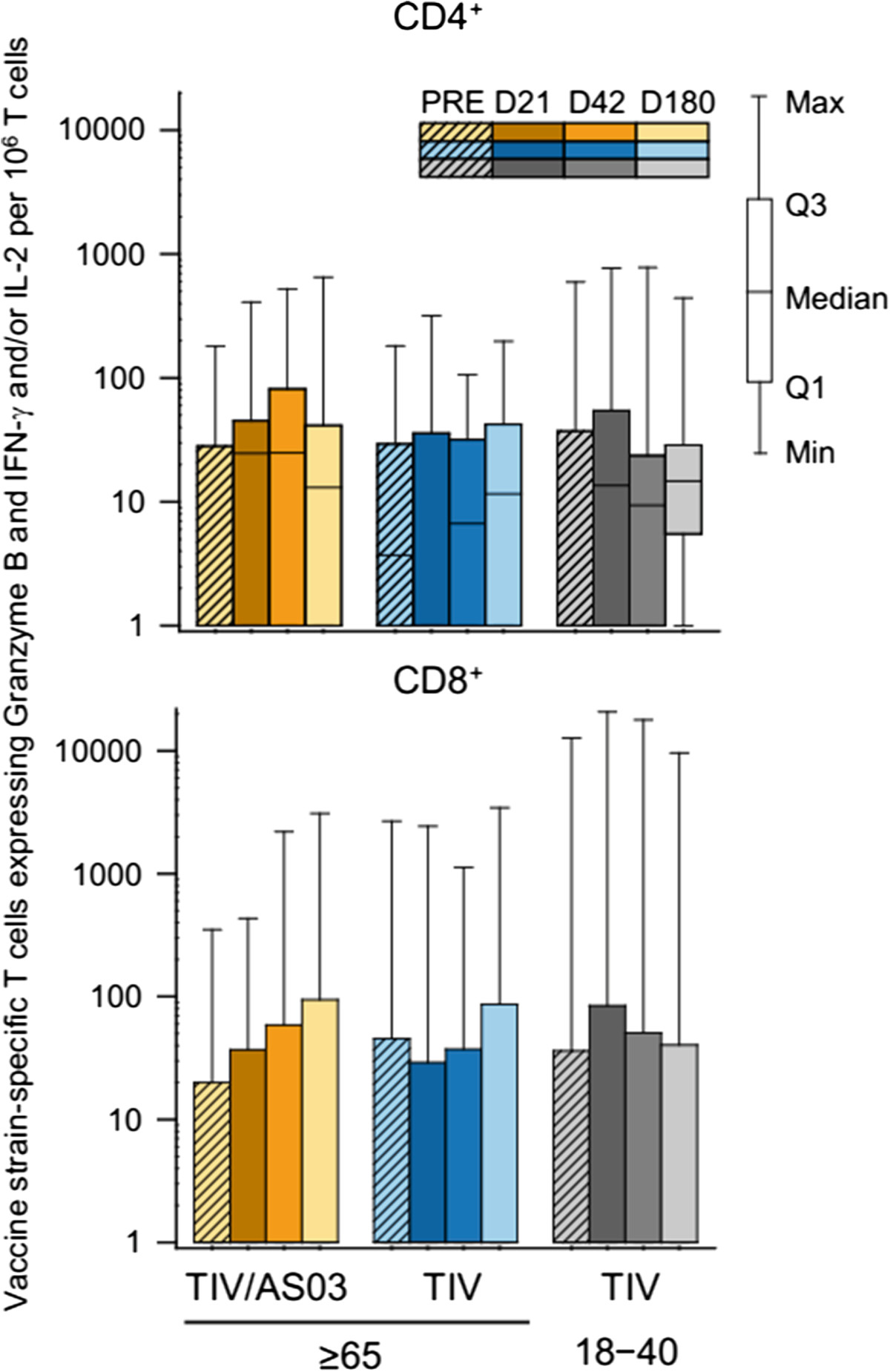
**Influenza-specific cytotoxic CD4** ^**+**^ **and CD8** ^**+**^ **T-cell responses to vaccination in the per protocol immunogenicity cohort.** Box and whisker plots describing the frequency of CD4^+^ or CD8^+^ T cells specific for the three (pooled) influenza vaccine strains and induced to express Granzyme B and IFN-γ and/or IL-2 (Spanish subjects only). For the TIV/AS03(≥65), TIV(≥65) and TIV(18–40) groups, N = 30–32, 35–36 and 24–25, respectively. The whiskers extend to the lowest (Min) and highest (Max) values; the box extends to the 1st quartile (Q1) and 3rd quartiles (Q3) in which the median is marked by a horizontal line.

No influenza-specific CD8^+^ T-cell response to vaccination was detected in any of the three groups (Figure 3). Vaccination also did not appear to affect the frequency of influenza-specific CD4^+^ or CD8^+^ T cells expressing Granzyme B and at least IFN-γ, and/or IL-2 (Figure 4).

There was no suggestion of bystander activation from the evaluation of the frequencies of CMV-specific CD4^+^ and CD8^+^ T cells expressing Granzyme B and at least IFN-γ, and/or IL-2 in CMV-seropositive subjects, because these frequencies appeared unaffected by vaccination (Additional file 1).

**Figure 5:**
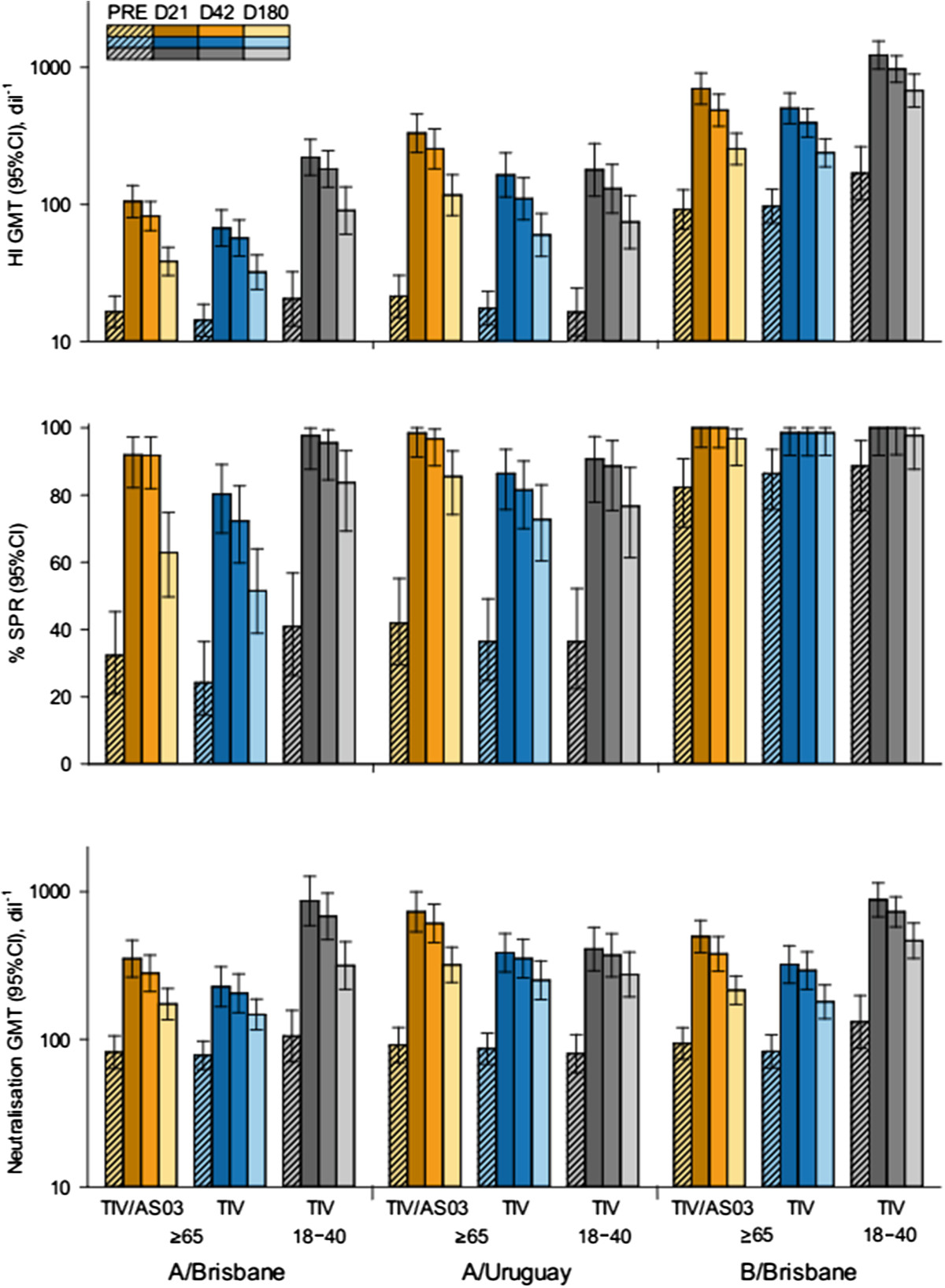
**Influenza-specific antibody responses to vaccination in the per protocol immunogenicity cohort.** Histograms describing geometric mean titres (GMT) for haemagglutinin inhibition, percentage seroprotection rates, and neutralisation GMTs at Day 0, Day 21, Day 42 and Day 180.

**Figure 6:**
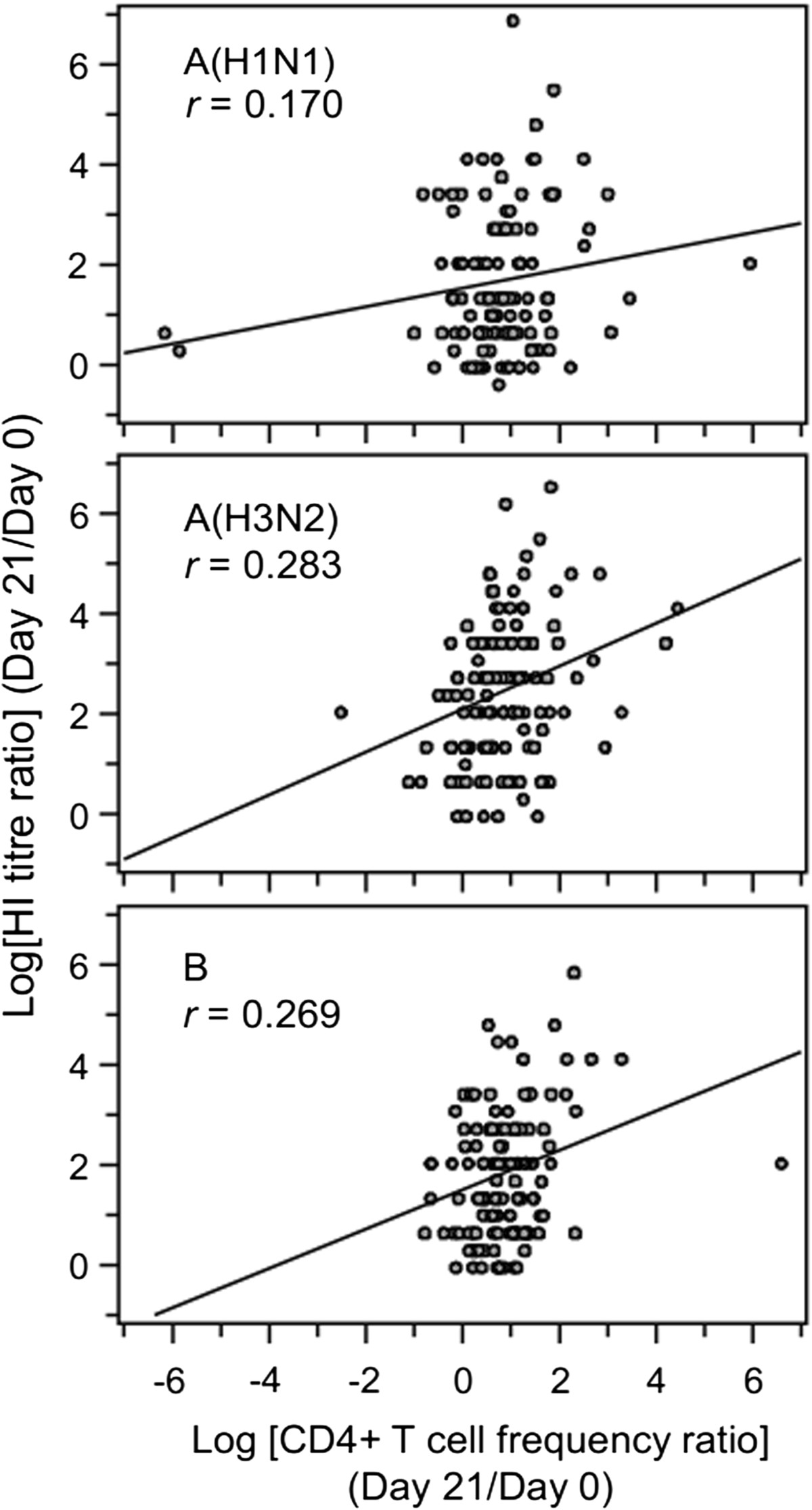
**Scatter plot comparisons of log-transformed values of fold-changes (Day 21 versus Day 0) of HI titres and frequencies of CD4** ^**+**^ **T cells expressing at least two immune markers specific for the three vaccine strains, in samples from all three groups of the Per Protocol cohort (N = 120, 120 and 118; upper, middle and lower graphs, respectively).** Pearson correlation coefficients (*r*) and related trend line are shown for each comparison.

Serum-HI titres (and SPRs and SCFs) and neutralisation titres, were determined before and after vaccination in each group (Figure 5 and Table 4). On Day 21, the SCFs for TIV/AS03(≥65) were 6.4, 15 and 7.6 against A/Brisbane(H1N1), A/Uruguay(H3N2) and B/Brisbane vaccine influenza strains, respectively. The corresponding SCFs for TIV(≥65) were 4.7, 9.4 and 5.2; and for TIV(18 - 40) were 11, 11 and 7.4. On Day 180, the range of SCFs for TIV/AS03(≥65) were 2.3–5.5, for TIV(≥65) were 2.2–3.4, and for TIV(18 - 40) were 4.0–4.6, suggesting a persistence of the antibody response in all treatment groups. No strong correlations were identified between the antibody and CD4^+^ T-cell responses to vaccination (i.e. the fold changes at Day 21 over Day 0) in all three groups considered together because Pearson r was less than 0.3 for each vaccine-specific set of responses (Figure 6).

**Table 4:** **HI seroconversion rates and seroconversion factors in response to vaccination**

Group	Day	N	A/Brisbane	A/Uruguay	B/Brisbane
			% seroconversion rate (95% CI)^a^		
TIV/AS03 (≥65)	21	62	58 (45–71)	84 (72–92)	71 (58–82)
	42	61	54 (41–67)	79 (66–88)	62 (49–74)
	180	62	26 (15–39)	58 (45–71)	35 (24–49)
TIV(≥65)	21	66	48 (36–61)	76 (64–86)	58 (45–70)
	42	65	38 (27–51)	68 (55–79)	54 (41–66)
	180	66	24 (14–36)	42 (30–55)	33 (22–46)
TIV(18-40)	21	43	67 (51–81)	77 (61–88)	67 (51–81)
	42	44	66 (50–80)	68 (52–81)	57 (41–72)
	180	43	53 (38–69)	46 (31–62)	46 (31–62)
			**Seroconversion factor (95% CI)** ^**a**^		
TIV/AS03 (≥65)	21	62	6.4 (4.5–9.0)	15 (11–22)	7.6 (5.6–10)
	42	61	5.1 (3.7–7.0)	12 (8.5–17)	5.4 (4.0–7.2)
	180	62	2.3 (1.8–3.0)	5.5 (4.1–7.3)	2.8 (2.2–3.4)
TIV(≥65)	21	66	4.7 (3.4–6.6)	9.4 (6.6–13)	5.2 (3.8–7.1)
	42	65	3.9 (2.8–5.4)	6.4 (4.7–8.6)	4.1 (3.1–5.4)
	180	66	2.2 (1.7–3.0)	3.4 (2.5–4.6)	2.5 (1.9–3.1)
TIV(18-40)	21	43	11 (6.3–18)	11 (7.2–18)	7.4 (4.7–12)
	42	44	8.9 (5.3–15)	7.9 (5.3–12)	5.8 (3.7–8.9)
	180	43	4.6 (2.9–7.2)	4.5 (3.0–6.9)	4.0 (2.8–5.8)

^a^Values reported at two significant figures.

TIV, trivalent influenza vaccine; TIV/AS03, AS03-adjuvanted trivalent influenza vaccine.

### Safety and reactogenicity

The most common injection site solicited symptom was pain and was more frequently reported in TIV/AS03(≥65) (62%) and TIV(18 - 40) (70%) groups than in the TIV(≥65) group (21%) (Table 5). Injection site solicited symptoms lasted for a mean duration of 2.3 - 3.5 days (TIV/AS03[≥65]), 2.0 days (TIV[18 - 40]), and 1.6 - 5.0 days (TIV[≥65]). No grade 3 injection site solicited symptoms were reported. Fatigue, headache and myalgia were the most frequently reported systemic solicited symptoms in the three groups. Most of the systemic solicited symptoms were more commonly reported in the TIV/AS03(≥65) and TIV(18 - 40) groups, than in the TIV(≥65) group. Grade 3 solicited symptoms were infrequently reported in all groups. The only grade 3 solicited systemic AEs reported were fever (1.4% in the TIV/AS03(≥65) group) and shivering (2.0% in the TIV(18 - 40) group). The percentages of subjects experiencing at least one unsolicited AE during the 21 day follow-up period were 19% (TIV/AS03[≥65]), 29% (TIV[≥65]) and 30% (TIV[18 - 40]). The percentages of subjects reporting grade 3 unsolicited AEs were 2.9%, 2.7% and 2.0% in these three groups, respectively. No grade 3 unsolicited AEs were considered as related to vaccination. Between Day 0 and Day 180, unsolicited medically-attended AEs were reported by 35%, 29% and 26% of subjects in the TIV/AS03(≥65), TIV(≥65), and TIV(18 - 40) groups, respectively, but none were considered related to vaccination. During the year follow-up, no pIMDs were reported. SAEs were reported by 17 subjects (5, TIV/AS03[≥65]; 11, TIV[≥65]; and 1, TIV[18 - 40]). One subject in the TIV/AS03 [≥65] group, aged 84 years, died from myocardial infarction at Day 256. None of the SAEs were considered related to vaccination.

**Table 5:** **Solicited injection site and systemic symptoms in response to vaccination**

Solicited symptom	% of subjects (95% CI) reporting symptoms, Day 0–6^a^			
	All grades	Grade 1	Grade 2	Grade 3
	**TIV/AS03(≥65) (N = 69)**			
***Injection site***				
Ecchymosis^b^	2.9 (0.4–10)	2.9 (0.4–10)	0.0 (0.0–5.2)	0.0 (0.0–5.2)
Pain^c^	62 (50–74)	52 (40–64)	10 (4.2–20)	0.0 (0.0–5.2)
Redness^b^	7.2 (2.4–16)	5.8 (1.6–14)	1.4 (0.0–7.8)	0.0 (0.0–5.2)
Swelling^b^	12 (5.1–22)	8.7 (3.3–18)	2.9 (0.4–10)	0.0 (0.0–5.2)
***Systemic***				
Arthralgia^cd^	20 (12–32)	15 (7.2–25)	5.8 (1.6–14)	0.0 (0.0–5.2)
Fatigue^cd^	32 (21–44)	17 (9.3–28)	15 (7.2–25)	0.0 (0.0–5.2)
Gastrointestinal^c^	8.7 (3.3–18)	5.8 (1.6–14)	2.9 (0.4–10)	0.0 (0.0–5.2)
Headache^c^	32 (21–44)	23 (14–35)	8.7 (3.3–18)	0.0 (0.0–5.2)
Myalgia^cd^	25 (15–37)	17 (9.3–28)	7.2 (2.4–16)	0.0 (0.0–5.2)
Shivering^cd^	16 (8.2–27)	12 (5.1–22)	4.3 (0.9–12)	0.0 (0.0–5.2)
Temperature^e^	2.9 (0.4–10)	1.4 (0.0–7.8)	0.0 (0.0–5.2)	1.4 (0.0–7.8)
	**TIV(≥65) (N = 73)**			
***Injection site***				
Ecchymosis^b^	1.4 (0.0–7.4)	1.4 (0.0–7.4)	0.0 (0.0–4.9)	0.0 (0.0–4.9)
Pain^c^	21 (12–32)	18 (9.8–29)	2.7 (0.3–9.5)	0.0 (0.0–4.9)
Redness^b^	0.0 (0.0–4.9)	0.0 (0.0–4.9)	0.0 (0.0–4.9)	0.0 (0.0–4.9)
Swelling^b^	1.4 (0.0–7.4)	1.4 (0.0–7.4)	0.0 (0.0–4.9)	0.0 (0.0–4.9)
***Systemic***				
Arthralgia^cd^	4.1 (0.9–12)	1.4 (0.0–7.4)	2.7 (0.3–9.5)	0.0 (0.0–4.9)
Fatigue^cd^	16 (8.8–27)	12 (5.8–22)	4.1 (0.9–12)	0.0 (0.0–4.9)
Gastrointestinal^c^	5.5 (1.5–13)	2.7 (0.3–9.5)	2.7 (0.3–9.5)	0.0 (0.0–4.9)
Headache^c^	9.6 (3.9–19)	8.2 (3.1–17)	1.4 (0.0–7.4)	0.0 (0.0–4.9)
Myalgia^cd^	11 (4.9–21)	9.6 (3.9–19)	1.4 (0.0–7.4)	0.0 (0.0–4.9)
Shivering^cd^	0.0 (0.0–4.9)	0.0 (0.0–4.9)	0.0 (0.0–4.9)	0.0 (0.0–4.9)
Temperature^e^	0.0 (0.0–4.9)	0.0 (0.0–4.9)	0.0 (0.0–4.9)	0.0 (0.0–4.9)
	**TIV(18-40) (N = 50)**			
***Injection site***				
Ecchymosis^b^	0.0 (0.0–7.1)	0.0 (0.0–7.1)	0.0 (0.0–7.1)	0.0 (0.0–7.1)
Pain^c^	70 (55–82)	46 (32–61)	24 (13–38)	0.0 (0.0–7.1)
Redness^b^	0.0 (0.0–7.1)	0.0 (0.0–7.1)	0.0 (0.0–7.1)	0.0 (0.0–7.1)
Swelling^b^	0.0 (0.0–7.1)	0.0 (0.0–7.1)	0.0 (0.0–7.1)	0.0 (0.0–7.1)
***Systemic***				
Arthralgia^cd^	8.0 (2.2–19)	6.0 (1.3–17)	2.0 (0.1–11)	0.0 (0.0–7.1)
Fatigue^cd^	42 (28–57)	36 (23–51)	6.0 (1.3–17)	0.0 (0.0–7.1)
Gastrointestinal^c^	12 (4.5–24)	12 (4.5–24)	0.0 (0.0–7.1)	0.0 (0.0–7.1)
Headache^c^	28 (16–43)	22 (11–36)	6.0 (1.3–17)	0.0 (0.0–7.1)
Myalgia^cd^	22 (11–36)	18 (8.6–31)	4.0 (0.5–14)	0.0 (0.0–7.1)
Shivering^cd^	4.0 (0.5–14)	2.0 (0.1–11)	0.0 (0.0–7.1)	2.0 (0.1–11)
Temperature^e^	0.0 (0.0–7.1)	0.0 (0.0–7.1)	0.0 (0.0–7.1)	0.0 (0.0–7.1)

^a^Values reported at two significant figures.

^b^Grade 1, >20– ≤ 50 mm; Grade 2, >50– ≤ 100 mm; and Grade 3, >100 mm.

^c^Grade 1, “easily tolerated” (“painful on touch” for injection site pain); Grade 2, “interferes with normal activity” (or “painful when limb is moved” for injection site pain); and Grade 3, “prevents normal activity” (or “considerable pain at rest” for injection site pain).

^d^Symptom was generalised/widespread.

^e^Grade 1, 38.0– < 38.5°C; Grade 2, 38.5– < 39.0°C; and Grade 3, 39.0– ≤ 40.0°C. No subjects reported temperature above 40°C.

CI, confidence interval; TIV, trivalent influenza vaccine; TIV/AS03, AS03-adjuvanted trivalent influenza vaccine.

The taking of medication during the study was reported by 64%, 53% and 54% of the subjects in the TIV/AS03(≥65), TIV(≥65), and TIV(18–40) groups, respectively. This included antipyretic medication, which was reported by 30%, 25% and 40% of the subjects in the TIV/AS03(≥65), TIV(≥65), and TIV(18–40) groups, respectively. No use of prophylactic antipyretic medication was reported.

## Discussion

The principal finding of this study is that TIV/AS03 is superior to TIV in that the inclusion of AS03 in TIV significantly enhanced vaccine antigen-specific CD4^+^ T-cell responses in ≥65 years-old adults on Day 21. This effect of TIV/AS03 was also evident for specific CD4^+^ T-cell responses against the three individual vaccine strains for the 180 day observation period.

Previous studies using the same methodology to measure T-cell responses have shown that AS03 formulated in A(H5N1) or A/California/7/2009(H1N1)pdm09 pandemic vaccines also induce stronger polyfunctional CD4^+^ T-cell responses than non-adjuvanted vaccine in adults aged 18 - 60 years [14, 31] and in older adults [21]. The similarity of the marker profile of the influenza specific CD4^+^ T cells elicited by the adjuvanted and non-adjuvanted vaccines indicates that AS03 affects primarily the magnitude of the specific CD4^+^ T-cell response rather than qualitative changes in the immune marker profile. This was also observed using different methodology in another study evaluating the effect on the immune response of the oil-in-water based adjuvant MF59 included in an A(H5N1) vaccine [39].

No influenza-specific CD8^+^ T-cell responses were detected after vaccination. Elsewhere, no specific CD8^+^ T-cell responses were detected with A(H5N1) and A(H1N1)pdm09 inactivated vaccines [14, 21, 31]. This does not necessarily indicate the absence of a CD8^+^ T-cell response. It could be that a CD8^+^ T-cell response occurred but peaked before Day 21, as suggested by our preliminary data showing CD8^+^ T-cell responses to A(H1N1)pdm09 vaccine on Day 7 (unpublished data). Alternatively, an optimal response *ex vivo* may require re-stimulation with a major histocompatibility complex-presented antigen by using infected cells instead of an inert split antigen. In another study on TIV, a CD8^+^ T-cell response was detected on Days 14 and 28 after vaccination using infected cell re-stimulation [40].

No changes in the frequencies of influenza-specific CD4^+^ T-cells expressing the cytolytic mediator Granzyme B (and IFN-γ, and/or IL-2) were detected during the study, suggesting that the vaccines had no effect on influenza-specific cytotoxic T cells. However, in other experiments, increased levels of Granzyme B activity have been observed in PBMC lysates (re-stimulated *ex vivo* with live influenza virus) in response to vaccination in older adults and which may have correlated with protection [27, 41]. The lack of a detectable effect on Granzyme B in our study may be due to differences in methodology, in particular the nature of the *ex vivo* stimulus or the exclusion in our assay of natural killer cells which contributed to the Granzyme B activity measured elsewhere [41]. In contrast, more than half the vaccine strain-specific T cells expressed IFN-γ. The frequencies of these IFN-γ^+^ CD4^+^ T cells appeared to increase post-vaccination, and they may have had the potential to exert some B-cell independent activity [42].

Although the study was not powered to compare humoral immune responses between groups, the results indicate an enhancing effect of AS03 on HI and neutralising antibody responses to TIV strains. Significantly enhanced antibody responses in older adults have been observed with AS03-adjuvanted A(H5N1) [21] and A(H1N1)pdm09 influenza vaccines [22]. The absence of substantial correlations between the magnitude of the antibody and CD4^+^ T-cell responses to vaccination was not unexpected. This absence has been observed before after single doses of seasonal trivalent A(H1N1)pdm09 and A(H5N1) pandemic vaccines [43–46]. In contrast, correlations have been identified after boosting with A(H5N1) prime/boost influenza vaccination regimens [39, 46], and by examining particular CD4^+^ T-cell phenotypes [47]. In this current study, memory T helper cells for the antibody response may have only represented a minor part of vaccine-strain specific T-cell response.

The incidence of some solicited symptoms was higher for TIV/AS03 compared with TIV in ≥65 year old subjects, but symptoms were mainly mild to moderate in nature. Furthermore, the incidences of most symptoms in the TIV/AS03(≥65) group were within the range for the TIV(18 - 40) group. The tolerability of TIV/AS03 was suggested by the similar incidences of unsolicited symptoms in the three groups and the lack of any clinically-observable safety concerns. These findings are consistent with a recent larger clinical study of AS03-adjuvanted seasonal influenza vaccine [30], and with clinical studies on AS03-adjuvanted A(H5N1) [21] and A(H1N1)pdm09 vaccines [22, 48] which also indicated that AS03-adjuvanted vaccines were well tolerated in older adults.

Other research into influenza immune responses [3, 26, 27, 49, 50] indicate that, in addition to humoral responses, T-cell responses could contribute to vaccine-mediated protection in older adults. Moreover, two recent studies have identified relationships between influenza disease severity and pre-existing disease strain-specific T-cells, in adults without pre-existing disease strain-specific antibodies [28, 29]. In a prospective study of adults (>18 years old) who became naturally-infected with A(H1N1)pdm09, disease severity was inversely correlated with pre-existing A(H1N1)pdm09-specific CD8^+^, but not CD4^+^, T cells [29]; whereas in a study in which adults (18–45 years old) were subjected to A(H3N2) or A(H1N1)-virus challenge, disease severity was inversely correlated with pre-existing challenge virus-specific CD4^+^, but not CD8^+^, T cells [28]. Although those contrasting results may be related to the different methodologies used (natural infection versus challenge; live virus versus peptides for identifying specific T cells with restimulation cell cultures) [29], the second study revealed a potential role of CD4^+^ T cells in protection, perhaps because these cells had direct cytotoxic activity or because they supported a CD8^+^ T-cell response through the expression of IFN-γ [28, 51]. Therefore the magnitude of the influenza-specific CD4^+^ T-cell response enhanced by AS03 in the seasonal vaccine may be directly relevant to increasing protection against disease in older adults.

## Conclusions

TIV/AS03 is superior to TIV in terms of vaccine antigen-specific CD4^+^ T-cell responses in ≥65 years-old adults on Day 21 post-vaccination. This positive effect of AS03 on the CD4^+^ T-cell response to influenza vaccine strains in older adults could confer benefit in protection against clinical influenza disease in this population.

## Endnote

^a^*Fluarix*™ is a trade mark of the GlaxoSmithKline group of companies.

## Electronic supplementary material

Additional file 1: CMV-specific cytotoxic CD4+ and CD8+ T-cell responses to vaccination in CMV-seropositive subjects of the Spanish subset of the per protocol immunogenicity cohort. Box and whisker plots describing the frequency of CD4+ or CD8+ T cells specific for CMV and induced to express Granzyme B and IFN-γ.and/or IL-2. For the TIV/AS03(≥65), TIV(≥65) and TIV(18–40) groups, N=23, 26 and 10, respectively. The whiskers extend to the lowest (Min) and highest (Max) values; the box extends to the 1st quartile (Q1) and 3rd quartiles (Q3) in which the median is marked by a horizontal line. (DOC 36 KB)

Below are the links to the authors’ original submitted files for images.

Authors’ original file for figure 1

Authors’ original file for figure 2

Authors’ original file for figure 3

Authors’ original file for figure 4

Authors’ original file for figure 5

Authors’ original file for figure 6

## Abbreviations

AS03: Adjuvant system 03
GSK: GlaxoSmithKline
pIMD: Potential immune mediated disease
AE: adverse event
CI: confidence interval
CMV: cytomegalovirus
GMF: geometric mean frequency
GMR: geometric mean ratio
GMT: geometric mean titres
GSK: GlaxoSmithKline
HA: Haemagglutinin
HI: haemagglutination-inhibition
PBMC: Peripheral blood mononuclear cell
pIMD: potential Immune Mediated Disease
r: Pearson correlation coefficient
SAE: serious adverse event
SBIC: Bayesian Information Criteria statistic
SCF: seroconversion factor
SCR: Seroconversion rate
SD: standard deviation
SPR: seroprotection rate
TIV: trivalent influenza vaccine
TIV/AS03: AS03-adjuvanted trivalent influenza vaccine
TVC: Total Vaccinated" cohort
US: United States.
