# Supplementary material for: Superior antigen-specific CD4+T-cell response with AS03-adjuvantation of a trivalent influenza vaccine in a randomised trial of adults aged 65 and older
Source: BMC Infect Dis. 2014 Jul 30;14:425. doi: 10.1186/1471-2334-14-425 (PMC4138369; doi:10.1186/1471-2334-14-425)
Supplement: Supplementary file 1 — Additional file 1: CMV-specific cytotoxic CD4+ and CD8+ T-cell responses to vaccination in CMV-seropositive subjects of the Spanish subset of the per protocol immunogenicity cohort. Box and whisker plots describing the frequency of CD4+ or CD8+ T cells specific for CMV and induced to express Granzyme B and IFN-γ.and/or IL-2. For the TIV/AS03(≥65), TIV(≥65) and TIV(18–40) groups, N=23, 26 and 10, respectively. The whiskers extend to the lowest (Min) and highest (Max) values; the box extends to the 1st quartile (Q1) and 3rd quartiles (Q3) in which the median is marked by a horizontal line. (DOC 36 KB) [file 12879_2014_3738_MOESM1_ESM.doc]

**Items to include when reporting a randomized trial in a journal abstract**

**Couch et al “Superior antigen-specific CD4+ T-cell response with AS03-adjuvantation of a trivalent influenza vaccine in a randomised trial of adults aged 65 and older”**

| **Item** | **Description** | **Reported on line number** |
| --- | --- | --- |
| Title | Identification of the study as randomized | Page 1: 2-3 |
| Trial design | Description of the trial design (e.g. parallel, cluster, non-inferiority) | Page 3: 6 |
| Methods |  |  |
| Participants | Eligibility criteria for participants and the settings where the data were collected | Page 3: 9, 6-7 |
| Interventions | Interventions intended for each group | Page 3: 9-11 |
| Objective | Specific objective or hypothesis | Page 3: 3-5 |
| Outcome | Clearly defined primary outcome for this report | Page 3: 11-14 |
| Randomization | How participants were allocated to interventions | Page 3: 9 |
| Blinding (masking) | Whether or not participants, care givers, and those assessing the outcomes were blinded to group assignment | Page 3: 6 |
| Results |  |  |
| Numbers randomized | Number of participants randomized to each group | - |
| Recruitment | Trial status | - |
| Numbers analysed | Number of participants analysed in each group | Page 3: 15-16 |
| Outcome | For the primary outcome, a result for each group and the estimated effect size and its precision | Page 3: 16-23 |
| Harms | Important adverse events or side effects | - |
| Conclusions | General interpretation of the results | Page 4: 1-3 |
| Trial registration | Registration number and name of trial register | Page 4: 4 |
| Funding | Source of funding | - |
