# Supplementary figures and images for: Superior antigen-specific CD4+T-cell response with AS03-adjuvantation of a trivalent influenza vaccine in a randomised trial of adults aged 65 and older
Source: BMC Infect Dis. 2014 Jul 30;14:425. doi: 10.1186/1471-2334-14-425 (PMC4138369; doi:10.1186/1471-2334-14-425)

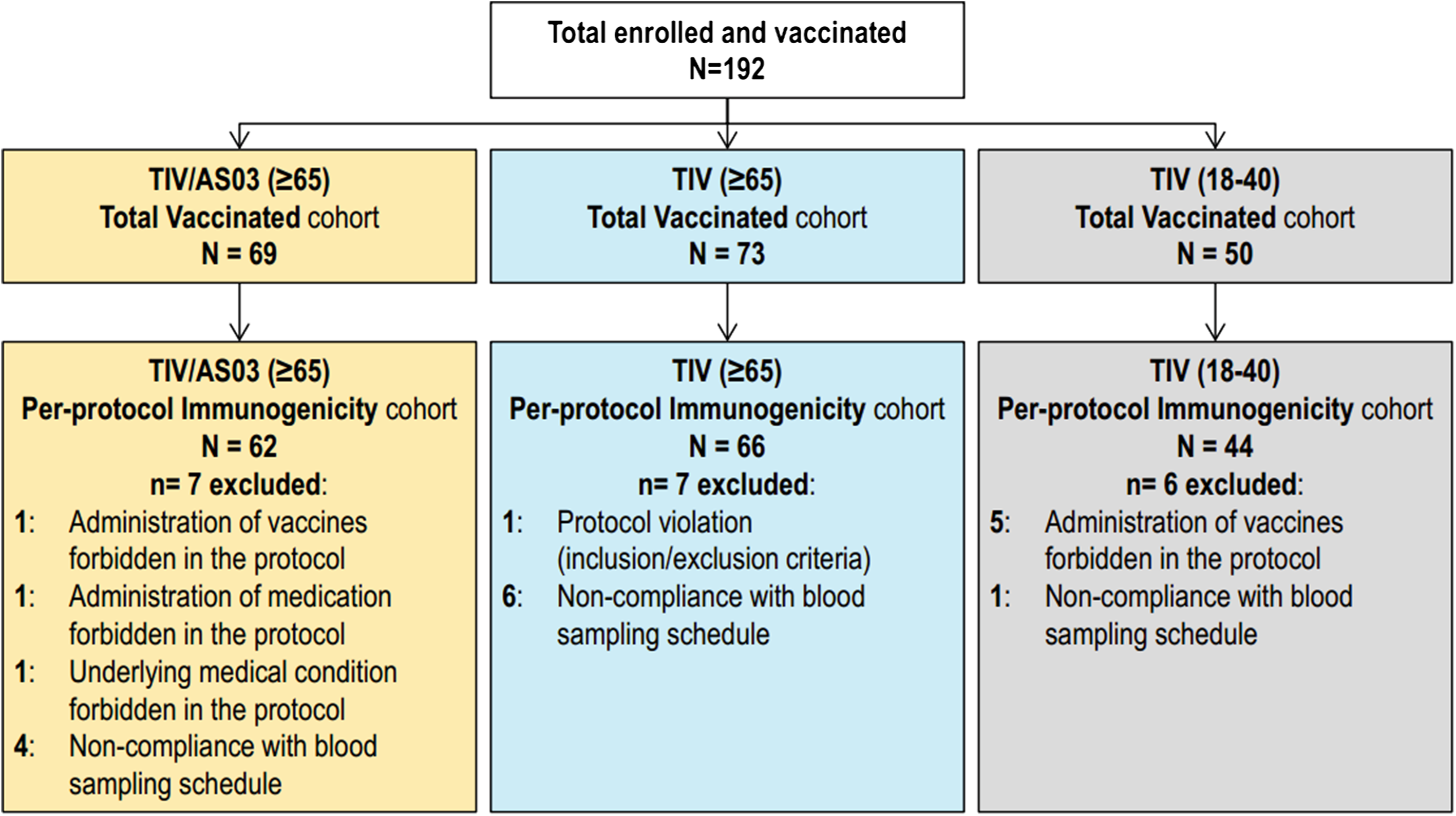

Supplement: Supplementary file 2 — Authors’ original file for figure 1 [file 12879_2014_3738_MOESM2_ESM.tif]

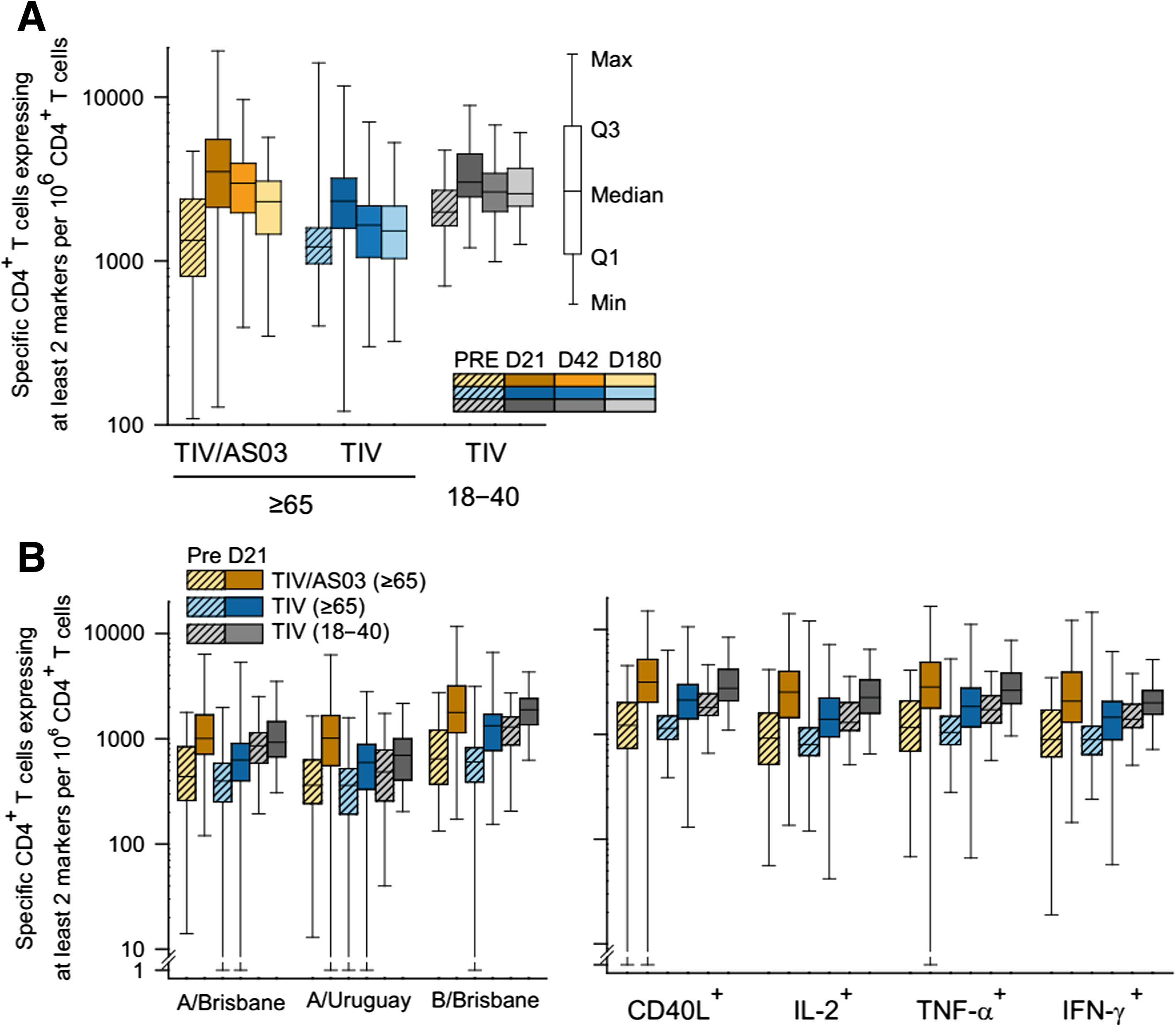

Supplement: Supplementary file 3 — Authors’ original file for figure 2 [file 12879_2014_3738_MOESM3_ESM.tif]

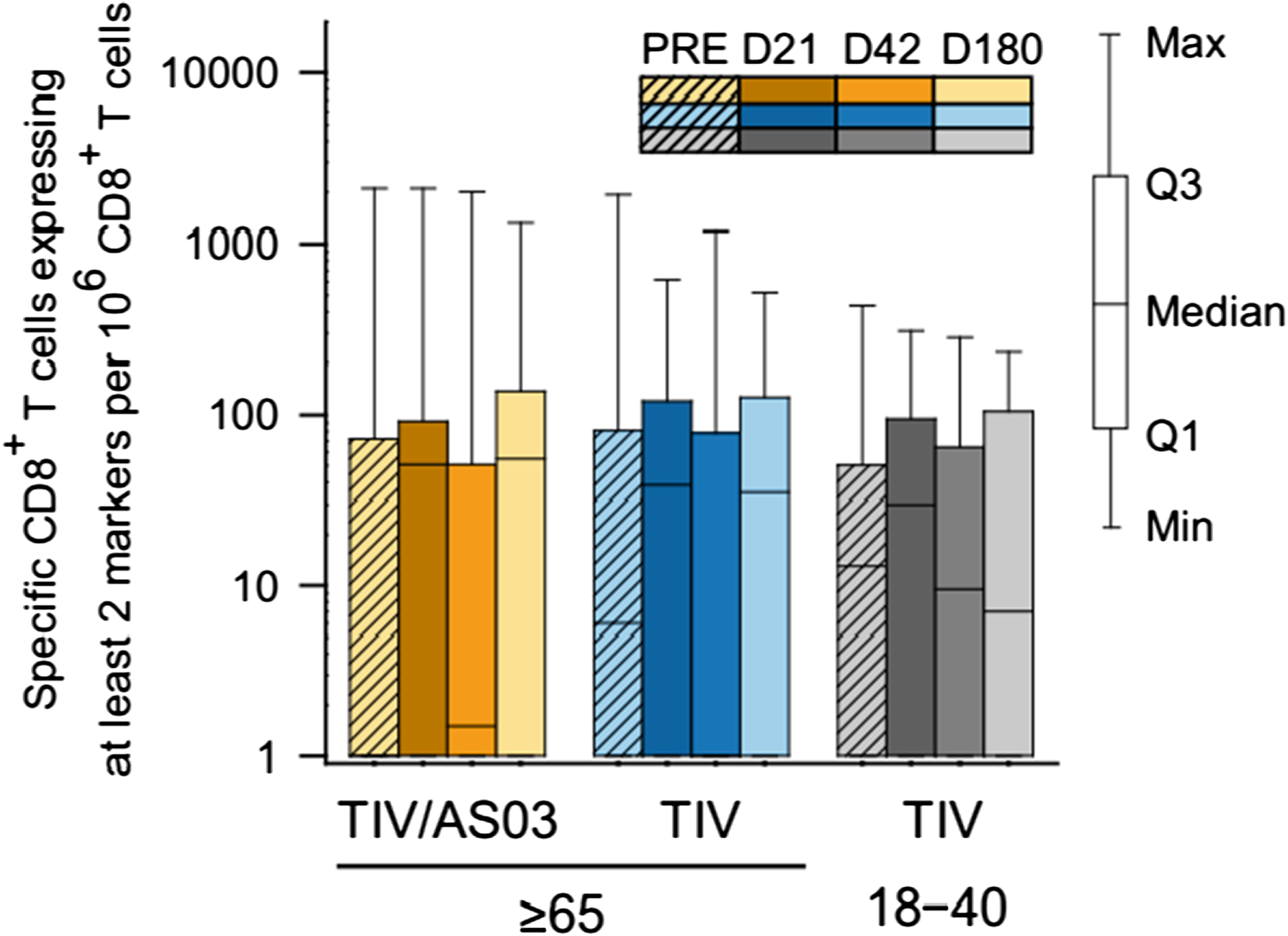

Supplement: Supplementary file 4 — Authors’ original file for figure 3 [file 12879_2014_3738_MOESM4_ESM.tif]

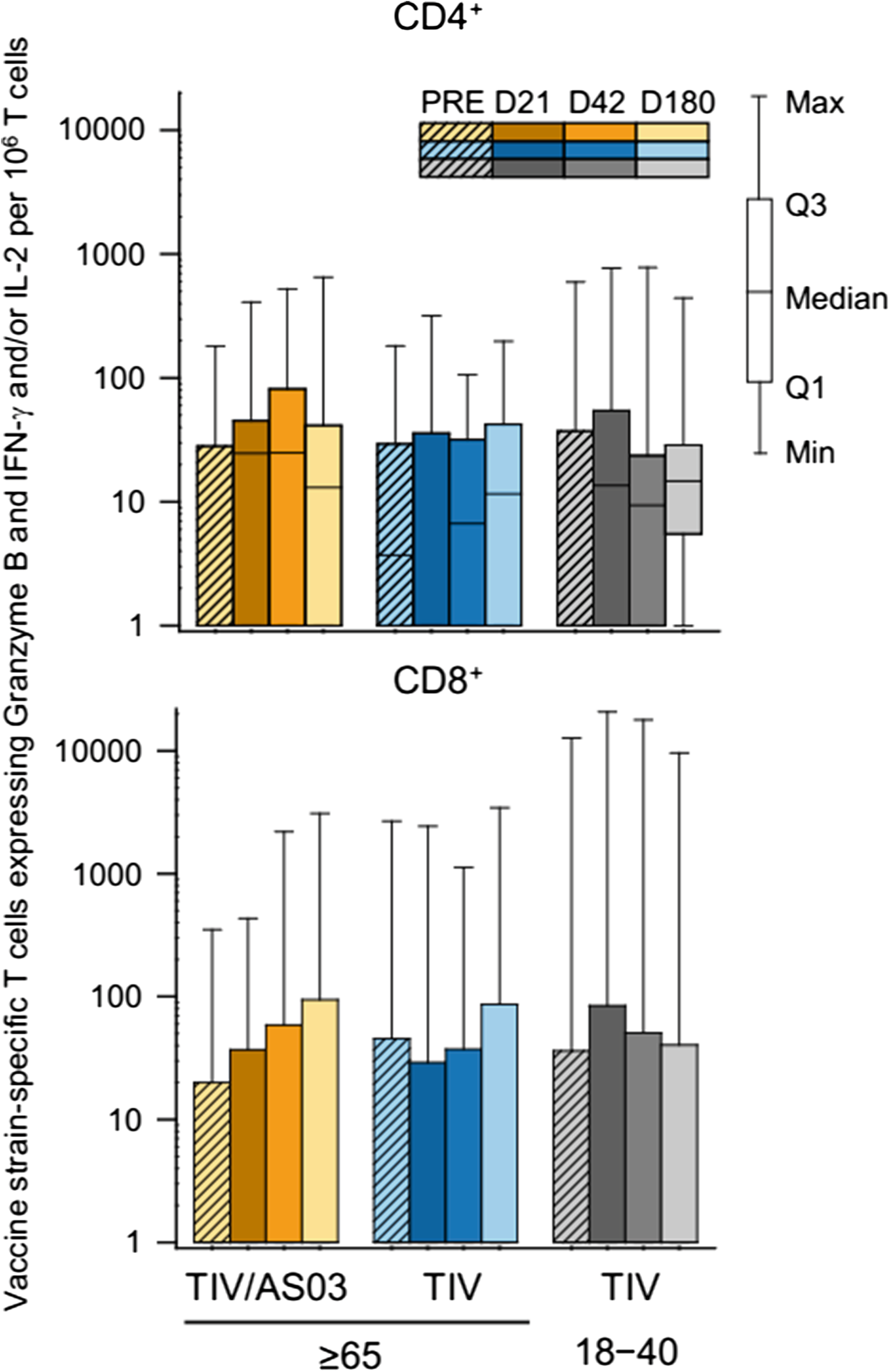

Supplement: Supplementary file 5 — Authors’ original file for figure 4 [file 12879_2014_3738_MOESM5_ESM.tif]

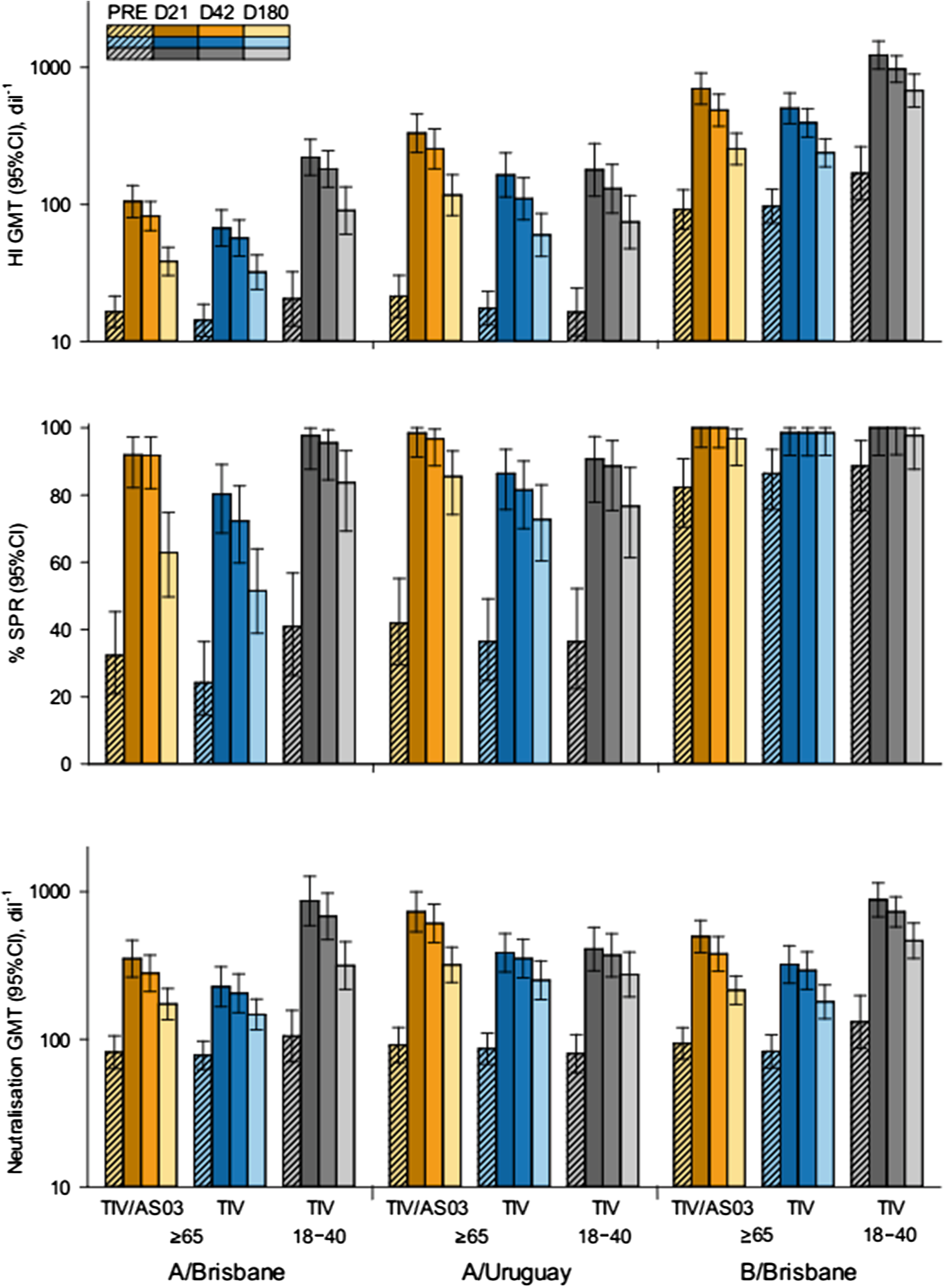

Supplement: Supplementary file 6 — Authors’ original file for figure 5 [file 12879_2014_3738_MOESM6_ESM.tif]

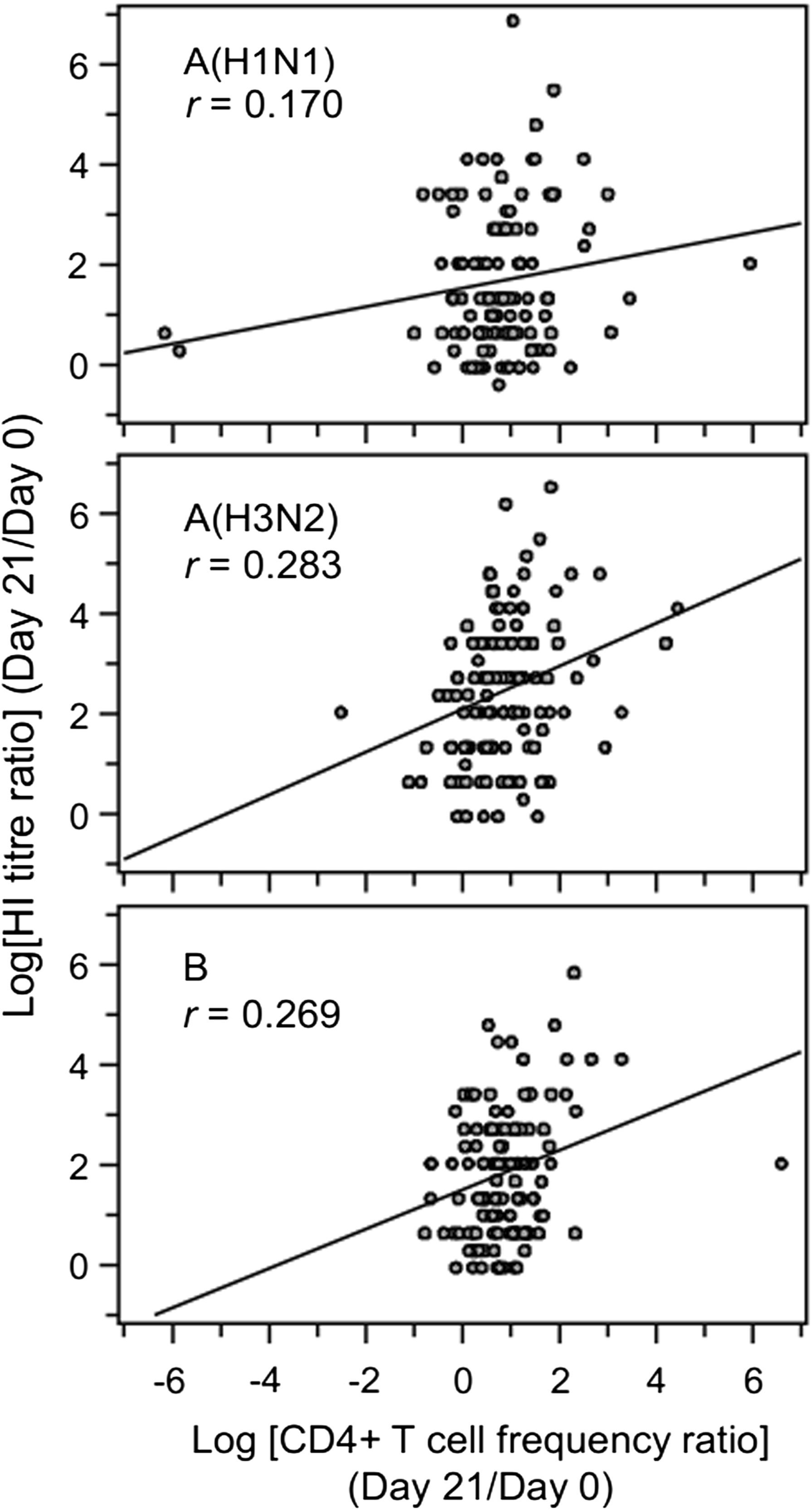

Supplement: Supplementary file 7 — Authors’ original file for figure 6 [file 12879_2014_3738_MOESM7_ESM.tiff]
